# Gingivitis Pathogenesis Involves Upregulation of Glycolysis and Citric Acid Cycle Activity Mediated by Bacterial Virulence Factors

**DOI:** 10.3390/ijms27125316

**Published:** 2026-06-12

**Authors:** Sancai Xie, Malgorzata Klukowska, Jiazhen Wang, Tom Huggins, Julie Ashe, Cheryl S. Tansky, Lijuan Li, Benjamin Circello, Niranjan Ramji, Donald J. White, Aaron R. Biesbrock

**Affiliations:** 1 Corporate Functions R&D, The Procter & Gamble Company, Mason, OH 45040, USA; wang.j.55@pg.com (J.W.); adellcrocket@gmail.com (T.H.); ashe.j@pg.com (J.A.); li.l.28@pg.com (L.L.); circello.bt@pg.com (B.C.); 2 Global Oral Care R&D, The Procter & Gamble Company, Mason, OH 45040, USA; klukowska.m@pg.com (M.K.); ramji.n@pg.com (N.R.); lamadental@outlook.com (D.J.W.); aaronbiesbrock@gmail.com (A.R.B.); 3 Global Baby, Feminine, Family Care Life Sciences, The Procter & Gamble Company, Cincinnati, OH 45224, USA; tansky.cs@pg.com

**Keywords:** stannous fluoride, adenosine triphosphate, malate dehydrogenase, triosephosphate isomerase, oral lavage, lipopolysaccharide, outer membrane vesicles, gingivitis

## Abstract

This research analyzed metabolomic and proteomic differences between participants with gingivitis (>20 bleeding sites) and generally healthy participants (≤3 bleeding sites) at baseline and 4 weeks post stannous fluoride (SnF_2_) dentifrice treatment. Sixty-two metabolites were different (*p* < 0.05) between groups at baseline. Forty cytokines were analyzed using immunoassays and a group of proinflammatory cytokines (IL-1α, IL-1β, TNF-α, SAA, ICAM-1, VCAM-1) was elevated in participants with gingivitis (*p* < 0.1) versus healthy gingiva at baseline, with C-reactive protein (*p* < 0.05) being significantly elevated. Proteomic analysis carried out in baseline oral lavage revealed four of the top hits (*p* < 0.0004) were central-metabolism-related: aldolase A, triosephosphate isomerase, lactate dehydrogenase, and malate dehydrogenase. Enzymatic assays confirmed the proteomic finding that malate dehydrogenase and triosephosphate isomerase activities were elevated in gingivitis samples; SnF_2_ dentifrice treatment reduced their activity. Collectively, 20 proteins with the lowest *p*-values in oral lavage appeared to be indicative of periodontal health, potentially forming the basis to cluster samples into healthy and unhealthy groups. A TLR-ATP biosensor model was established and demonstrated that microbial virulence factors induced the observed changes in oral lavage. Combined findings suggest gingivitis involves upregulation of host cell bioenergetic processes involving enzymatic activity in the glycolysis and citric acid cycle pathways.

## 1. Introduction

Periodontal diseases are a group of chronic inflammatory conditions in the periodontium that initially present as gingivitis, a bacterial-induced inflammation of the marginal and attached gingiva [1,2,3]. The clinical symptoms of gingivitis include redness, edema, and bleeding at the gingival margin. The prevalence of gingivitis in adult populations is high, with studies estimating it to be 65% or higher [4,5,6]. Treatment and management of gingivitis is of clinical importance, because left untreated it can progress to periodontitis, which includes loss of clinical attachment, recession, increased tooth mobility, and tooth loss. The absence of bleeding on probing over repetitive dental examinations has been shown in longitudinal studies to be a reliable predictor for the maintenance of periodontal health, as measured by lack of gingival attachment and tooth loss [7,8,9,10,11].

The microbial etiology of gingivitis was first established by the landmark studies of Löe et al. in 1965, where researchers demonstrated that gingivitis could be induced in healthy patients through the cessation of daily oral hygiene, known as the Experimental Gingivitis [EG] model [12]. The researchers showed that the gingivitis developed in EG could be rapidly reversed by thorough removal of dental plaque from the teeth and gingival margin. Later, culturing techniques detailed the microbial succession that occurred in the plaque biofilm during the EG phase, with a shift from a *Streptococcus*-dominated biofilm to an *Actinomyces* biofilm as the plaque aged over sans hygiene phase [13]. Importantly, the bacterial species *Actinomyces viscosus* and *Bacteroides melaninogenicus* were elevated significantly at gingival sites that bled, suggesting a strong association [13,14]. The development of diverse microbial communities during the development of EG has been confirmed by additional culturing studies [15,16] as well as by more contemporary molecular biology probes including 16S RNA [17,18]. It is now generally accepted that in chronic gingivitis/periodontal disease, plaque microbiology transforms with the development of significantly greater and varied Gram-negative anaerobes. The microbiological profile shows that gingivitis sites (bleeding) harbor more Gram-negative anaerobic bacteria in plaque compared to non-gingivitis sites (non-bleeding) [19,20]. The proliferation of anaerobes in mature plaque is theorized to result from various environmental features of thickened dental plaques, with the decreased oxygen tension playing a prominent role [21,22]. This maturation process has been described by Marsh as an example of an ‘ecological catastrophe’ and the plaques which develop are described by microbiologists as dysbiotic [21,22]. The microbiological profile of dysbiotic plaque includes a diverse group of Gram-negative anaerobes and obligate anaerobes, including the species *Fusobacterium nucleatum*, *Porphyromonas gingivalis*, *Treponema denticola*, and *Tannerella forsythia* amongst others. The combination of ‘*Porphyromonas gingivalis*, *Tannerella forsythia* and *Treponema denticola*’ has been defined by Socransky and co-workers as the ‘red complex’ owing to their proposed association with the development of periodontal disease [23,24,25].

It is now appreciated that the polymicrobial communities in dysbiotic dental plaques contain or produce a variety of ‘virulence factors’ which may contribute to activation of the innate immune response, stimulate release of proinflammatory cytokines and proteases, and trigger inflammatory disease development [26,27]. Gingivitis is a microbial-mediated inflammatory disease of the gingiva, which is a precursor to the more advanced inflammatory disease of periodontitis. In periodontitis, the unresolved inflammatory lesion exacerbates, leading to microbial-triggered host-mediated destruction of connective tissue and bone. Some virulence factors are associated with metabolism of bacteria and host tissue in the dental plaque, including, for example, production and secretion of various enzymes (e.g., gingipains) [28,29], protein degradation debris [27], various non-protein metabolites such as volatile sulfur species [30], and short-chain carboxylic acids [31]. Other virulence factors of anaerobic dental plaques are directly associated with the molecular composition of bacteria themselves such as lipopolysaccharides (LPS), lipoteichoic acid (LTA) and peptidoglycans, substances found in the cell walls of the microbes and secreted in outer membrane vesicles (OMVs) from certain species [32,33,34]. The LPS, LTA and peptidoglycan molecular structures of oral pathogens (highlighted above) are known as ‘pathogen-associated molecular patterns’ (PAMPs). The PAMPs are detected and recognized by specialized receptors called pattern recognition receptors (PRRs) which are ubiquitously expressed in numerous immune and epithelial cells [35,36]. The PRRs play a crucial role in the innate immune system to fight against a variety of microbial pathogens including bacteria, viruses, parasites, fungi, and protozoa [35,36]. The best known PRRs are the Toll-like receptors (TLRs). They are single, membrane-spanning, non-catalytic receptors, evolutionarily conserved and homologues of the Drosophila Toll-like protein [35,36]. In the periodontium, TLRs have been detected in various leukocytes traversing the periodontal tissues, gingival epithelial cells, gingival fibroblasts, endothelium, periodontal ligament fibroblasts, osteoblasts, osteoclasts and cementoblasts [36]. Based on available evidence, TLR4 and TLR2 would appear to be associated with the development of inflammatory responses to oral pathogens and the development of chronic inflammation associated with periodontal disease [36,37,38]. LPS, a component of the outer membrane of Gram-negative bacteria, is recognized by TLR4, while peptidoglycans, a major component of bacterial cell walls, are produced by both Gram-positive and Gram-negative bacteria and bind to TLR2 [38]. Both TLR2 and TLR4 are found on the surface of innate immune cells, such as neutrophils, monocytes, and macrophages [36,37,38]. When microbial virulence factors bind to TLR2 and TLR4, they trigger a signaling cascade that results in the production of cytokines, a signature of activating immune responses. Using gene signaling reporter assays, we found that both TLR2 ligands (peptidoglycans and LTA) and TLR4 ligands (LPS) are more abundant in the subgingival plaques of bleeding sites than in non-bleeding sites [39,40].

Microbial infection and proliferation as well as the innate immune response initiate changes in gene expression, protein production and metabolism of gingival tissue. The released proteins and metabolites from dental plaque and gingival tissues are accumulated in saliva, which is mainly secreted by the salivary glands, together with exudate from gingival sulcus and oral mucosa. Because of this, researchers have suggested that saliva might be a good substrate for evaluations of the features of microbial dysbiosis and periodontal inflammation. A recent study compared periodontitis biomarkers in saliva, oral lavage, and gingival crevicular fluid, revealing that oral lavage offered the highest accuracy in distinguishing between gingival health and periodontitis [41]. Analysis of saliva typically includes the use of oral lavage. Techniques applied to oral lavage samples include Nuclear Magnetic Resonance (NMR) [42,43] and mass spectrometry [44,45,46]. Complementing these techniques, saliva samples have also been extensively studied using immunoassays [47,48] and proteomics [49,50,51,52,53]. Metabolites identified in saliva have included succinate, lactate, propionate, butyrate, indolepropionic acid, cadaverine, putrescine, arginine, histidine, and ornithine. In addition, saliva analysis has revealed elevated protease activities and elevated levels of degradation products of fatty acids and carbohydrates and especially glycolysis and citric acid cycles. Signatures of oxidative stress have also been observed to be exacerbated. Proinflammatory cytokines (IL-1β, IFNγ, IL-6, VEGF, TNFα) were often reported to increase in the saliva of patients with gingivitis and periodontitis [47,48]. In addition, other proteins, such as CSF-1, S100A8/A9, S100A12, MMP-8, and HGF, were significantly elevated in saliva from periodontitis and gingivitis patients in comparison to healthy individuals [47,48].

There has been increased global commercialization and distribution of SnF_2_ dentifrices in the last 20 years. These dentifrices now represent the majority of therapeutic toothpaste sold with proven efficacy for the treatment of gingivitis [54]. Previously, a clinical study was conducted in which 20 participants with gingival health (low bleeding) and 20 with gingivitis (high bleeding) were enrolled. All participants carried out brushing with a Sn-based dentifrice for 4 weeks. Clinically, both bleeding and inflammation scores were improved for both groups at week 4 [39]. Microbial virulence factors in the subgingival plaques were examined by TLR reporter assays and were shown to be reduced at week 4 in participants using SnF_2_ dentifrice [40]. Metabonomic analyses of oral lavage samples showed marked changes in the total metabolic profile and that short-chain fatty acids (propionic acid and butyric acid) were also reduced in the saliva of these participants [43]. The metabolite analysis carried out in the former study was limited due to the limitations of the NMR diagnostic method applied to the saliva sample. Importantly, samples from this previous study were available, having been stored under conditions preserving their composition. The present supplemental analysis of the oral lavage specimens examined the difference in metabolomic and proteomic profiles between patients with gingivitis (unhealthy gingiva) and generally healthy participants (healthy gingiva) at baseline and to determine the effects of SnF_2_ dentifrice treatment on metabolomic and proteomic profiles following 4 weeks of treatment. Metabolites in the oral lavage samples were analyzed using Metabolon’s global biochemical profiling mass spectrometry procedures [55]. Oral lavage samples were also analyzed for proteomics using both immunoassay-based and aptamer-based methods. Lastly, findings from the metabolomic analysis prompted the development of a novel TLR–adenosine triphosphate (ATP) biosensor model in a novel cell line that was used to determine the role of bacterial toxin in stimulating TLR-mediated ATP production as a putative step in the initiation of an inflammatory response, and the role of SnF_2_ in suppressing TLR-mediated ATP production.

## 2. Results

### 2.1. Clinical Observations, Microbial Composition and Virulence Assessments from Original Study: Effects of Four Weeks of SnF_2_ Dentifrice Use

Baseline demographic and clinical data is summarized in Supplementary Table S1. As reported previously, clinical assessments revealed statistically significant reductions in modified gingivitis index (26%) and gingival bleeding index (53%) associated with four weeks of SnF_2_ dentifrice treatment, with efficacy documented in unhealthy gingiva as well as healthy gingiva participants [39]. Analysis was carried out on supra- and subgingival samples of plaque, as well as oral lavage samples from baseline and week 4. Bacterial culture analysis of both supragingival and subgingival plaque samples showed statistically significantly reduced numbers of Gram-negative anaerobes following SnF_2_ dentifrice treatment in both high- and low-disease groups. Subgingival plaque samples also exhibited significantly reduced virulence in both groups as measured in receptor-based assay in TLR4-transfected HEK293 cells using a SEAP alkaline phosphate reporter as a marker of Toll receptor activation [39]. Metabonomic assessments of saliva carried out by NMR analysis of oral lavage samples demonstrated statistically significant reductions in propionic and butyric acid in both groups, short-chain fatty acids associated with SnF_2_ dentifrice use, along with significant overall changes in metabolite profiles [43]. Clinical results are summarized in Supplementary Figure S1. These observations support the hypothesis that the effectiveness of SnF_2_ dentifrice in improving gingival health is linked to the reduction in Gram-negative bacteria and microbial virulence factors in subgingival plaques. The results that follow present additional observations regarding how bacterial virulence factors alter the metabolism of gingival cells, disrupt cellular bioenergetics, and cause oxidative stress.

### 2.2. Metabolomic Analysis of Oral Lavage (Metabolon Analysis): Baseline Comparisons Between Unhealthy and Healthy Gingiva Groups

**Figure 1:**
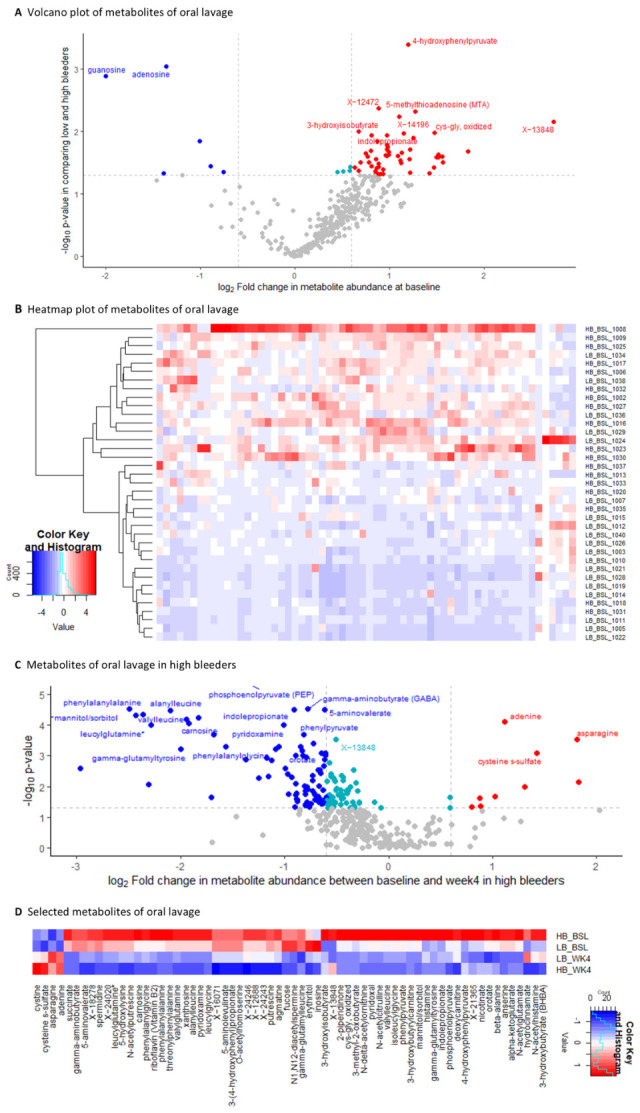
Concentrations of metabolites in oral lavage between low and high bleeders at baseline (**A**,**B**) and metabolites in oral lavage after 4 weeks of treatment with stannous fluoride dentifrice (**C**,**D**). (**A**). Volcano plot comparing metabolites in oral lavage between low and high bleeders at baseline. Metabolites are grouped into four categories based on *p*-values (Y-axis) and fold-change (X-axis): Dark blue: *p* ≤ 0.05 and log2 fold-change < −0.6; Grey: *p* > 0.05; Teal: *p* ≤ 0.05 and log2 fold-change between −0.6 and 0.6; Red: *p* ≤ 0.05 and log2 fold-change > 0.6. (**B**). Heatmap clustering oral lavage samples based on 62 metabolites that differed significantly (*p* ≤ 0.05) between low and high bleeders at baseline. HB represents high bleeder, LB represents low bleeder, BSL represents baseline and the four digits are the participant code. (**C**). Volcano plot comparing metabolites in oral lavage between baseline and week 4 in high bleeders. Metabolites with *p* ≤ 0.001 are labeled. Red: *p* ≤ 0.05 and log2 fold-change > 0.6; Dark blue: *p* ≤ 0.05 and log2 fold-change < −0.6; Grey: *p* > 0.05; Teal: *p* ≤ 0.05 and log2 fold-change between −0.6 and 0.6. (**D**). Heatmap comparing metabolites in oral lavage at baseline and week 4 in both low and high bleeders. Metabolites shown in the heatmap are significantly different with *p* ≤ 0.01 between baseline and week 4 in the high-bleeder group.

Metabolites were compared between low- and high-bleeding participants at baseline (Figure 1A,B). A volcano plot was used to compare metabolites between high and low bleeders labeled in the graph (*p* < 0.011). Two purines—adenosine and guanosine—were observed to be significantly lower in the oral lavage of high bleeders compared to low bleeders. Four identified metabolites were significantly elevated in the high bleeders’ oral lavage (*p* < 0.011), including 5′-methylthioadenosine (MTA), oxidized cys-gly, 3-hydroxyisobutyrate, and indolepropionate. Sixty-two metabolites showed a difference at *p* ≤ 0.05 between low and high bleeders at baseline. The results were used to generate clustering and a heatmap (Figure 1B). Most high bleeders (11/18) were clustered at the top of the heatmap while most of the low bleeders (14/19) were clustered at the bottom. This suggests that 62 metabolites could classify healthy and unhealthy gingiva.

### 2.3. Changes in Metabolite Levels in Oral Lavage: Effects of Four Weeks of SnF_2_ Dentifrice Use

Metabolite levels in oral lavage were compared between baseline and week 4 in unhealthy gingiva (Figure 1C,D, Supplementary Table S2). Metabolites with *p* ≤ 0.001 were labeled in a volcano plot (Figure 1C). Significant changes were observed in the metabolic profile between baseline and week 4. Many dipeptides drastically declined after 4 weeks of treatment, indicating a reduction in tissue damage due to elevated tissue protease activities with inflammation. Two metabolites, oxidized cys-gly and indolepropionate, which were significantly elevated in unhealthy gingiva at baseline, decreased at week 4. Notably, two metabolites in the citric acid cycle, α-ketoglutarate and succinate, also decreased remarkably with SnF_2_ treatment (*p* < 0.001). A heatmap comparing metabolites in oral lavage at baseline and week 4 is shown in Figure 1D for both healthy and unhealthy gingiva. Significant shifts were associated with SnF_2_ dentifrice treatment compared to baseline; metabolites shown in the heatmap were significantly different (*p* ≤ 0.01) in the unhealthy gingiva group.

### 2.4. Biomarker 40-Plex: Baseline Comparisons Between Unhealthy and Healthy Gingiva Groups

**Figure 2:**
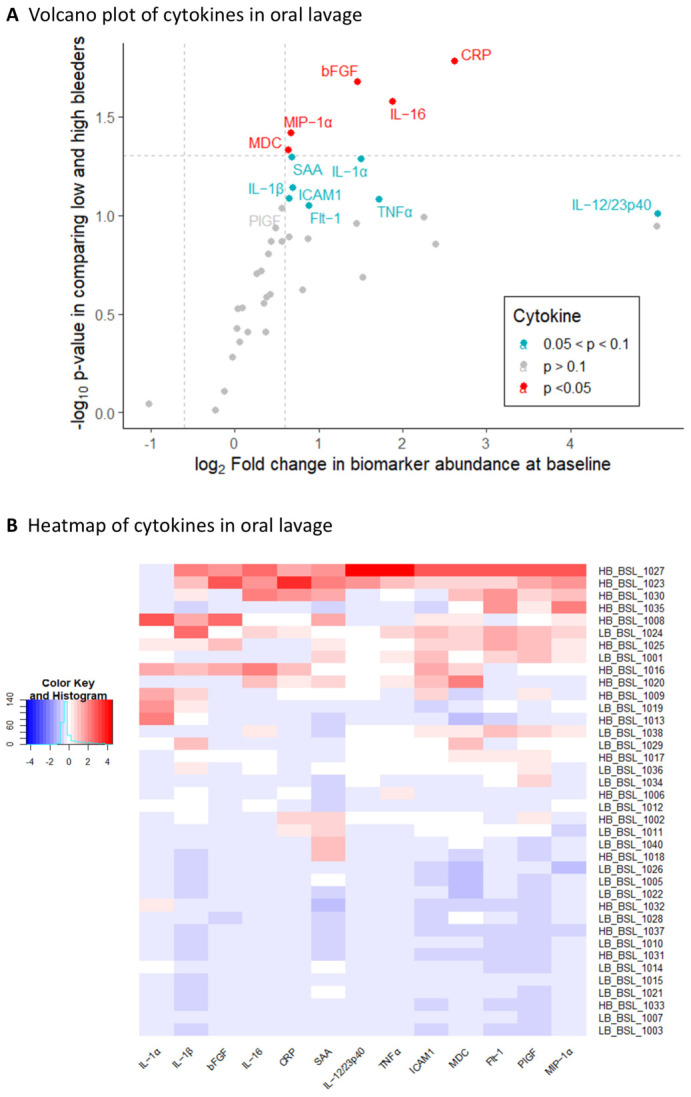
Antibody-based analyses of 40-plex protein biomarkers in oral lavage. (**A**). The 40-plex protein biomarkers were measured using antibody-based immune assays and compared in a volcano plot. The fold-change on the x-axis represents the ratio of high bleeders to low bleeders at baseline. Red dots highlight protein biomarkers with *p* < 0.05, while teal dots indicate *p* < 0.1, grey dots *p* > 0.1. (**B**). Thirteen protein biomarkers with *p*-values < 0.1 were used to construct a heatmap, displaying the differences in oral lavage between low and high bleeders at baseline. The biomarkers include bFGF (basic fibroblast growth factor), CRP (C-reactive protein), Flt-1 (vascular endothelial growth factor receptor 1), ICAM-1 (intercellular adhesion molecule-1), IL-1α (interleukin 1 α), IL-1β (interleukin 1 β), IL-12/23p40 (interleukin 12/interleukin 23 p40), IL-16 (interleukin 16), MDC (macrophage-derived chemokine), MIP-1α (macrophage inflammatory protein-1 α), PIGF (placental growth factor), SAA (serum amyloid A), and TNF-α (tumor necrosis factor α). HB represents high bleeder, LB represents low bleeder, BSL represents baseline, and the four digits are the participant code.

Five biomarkers (CRP, bFGF, IL-16, MIP-1α, MDC) in oral lavage were significantly different between healthy and unhealthy gingiva (*p* < 0.05, Figure 2A,B) at baseline. CRP was elevated in unhealthy gingiva compared to healthy gingiva. Several proinflammatory cytokines and biomarkers (IL-1α, IL-1β, TNF-α, SAA, ICAM-1, Fit-1, PIGF, IL-12/23p40) were directionally increased in unhealthy gingiva participants as well (*p* < 0.1). The 13 biomarkers with *p*-values < 0.1 were used to construct a baseline heatmap plot (Figure 2B), where unhealthy gingiva subjects clustered at the top of the graph, while healthy gingiva subjects converged at the bottom.

### 2.5. Proteomic Analysis of Oral Lavage Using Aptamer-Based Reagents: Baseline Comparisons Between Unhealthy and Healthy Gingiva Groups

**Figure 3:**
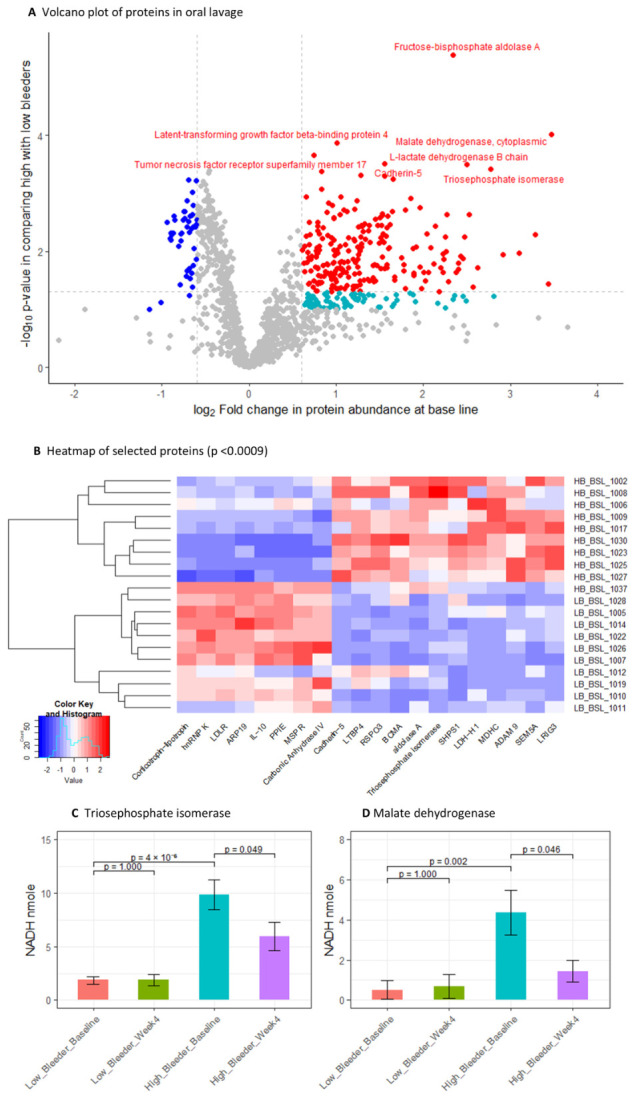
Difference in protein abundance between low- and high-bleeder groups in oral lavage at baseline (**A**,**B**) and changes in enzymatic activities in oral lavage after treatment with stannous fluoride (**C**,**D**). Proteins were analyzed using aptamer-based reagents at SomaLogic Inc. (**A**). All proteins were used to create a volcano plot, with the ratios of protein abundance between high and low bleeders on the x-axis. Proteins are grouped into four categories based on *p*-values (Y-axis) and fold-change (X-axis): Dark blue: *p* ≤ 0.05 and log2 fold-change < −0.6; Grey: *p* > 0.05; Teal: *p* ≤ 0.05 and log2 fold-change between −0.6 and 0.6; Red: *p* ≤ 0.05 and log2 fold-change > 0.6. The seven proteins with the lowest *p*-values are labeled in the plot. (**B**). Proteins were selected based on *p* < 0.0009 to construct a heatmap and dendrogram. HB_ represents high bleeder, LB represents low bleeder, BSL represents baseline, and the four digits are the participant code. (**C**). Triosephosphate isomerase and (**D**). malate dehydrogenase activities were measured and expressed as NADH nmole. Their activities were compared between baseline and four weeks after treatment with stannous fluoride dentifrice within both low- and high-bleeder groups. Additionally, the differences in their activities between low- and high-bleeder groups were compared at baseline.

The subset of ten oral lavage samples from both healthy and unhealthy gingiva at baseline were selected based on metabolic profiling (Figure 1) and clinical evaluation, then sent to Somalogic for detailed proteomic analysis. A total of 1310 proteins were identified and measured using aptamer-based reagents. The difference between healthy and unhealthy gingiva was compared in the volcano plot (Figure 3A; values in Supplementary Table S3).

Seven proteins, exhibiting *p*-values below 0.0004, were observed to be more abundant in the oral lavage of individuals with unhealthy gingiva compared to those with healthy gingiva. Four of these proteins are recognized as being central to cellular glycolysis (aldolase A, triosephosphate isomerase, LDH-H1) and the citric acid cycle (MDHC, malate dehydrogenase cytoplasm), potentially suggesting their involvement in altered metabolism and ATP generation in this context. To investigate whether these protein biomarkers could offer insights into clustering healthy and unhealthy gingiva subjects, a heatmap was generated using the 20 proteins with the lowest *p*-values (*p* < 0.0009) (Figure 3B). It is important to consider that in this preliminary analysis, involving a relatively small sample size of 20 subjects, all individuals designated as healthy consistently formed a distinct cluster. Interestingly, one participant from the unhealthy gingiva cohort (HB_BSL_1037) was grouped alongside the healthy gingiva subjects in the dendrogram, which may indicate a degree of shared protein biomarker distribution with the healthy gingiva group. A cross-reference to Figure 1B further supported this observation, as the same participant (HB_BSL_1037) was also similarly clustered with the healthy gingiva group in the dendrogram derived from 62 selected metabolites.

### 2.6. Enzymatic Activities: Baseline Comparisons Between Unhealthy and Healthy Gingiva Groups

Triosephosphate isomerase and MDHC were notably elevated in oral lavage from high-bleeding participants compared to low bleeders at baseline in the SomaLogic proteomic analyses (Figure 3). To expand and confirm these results, sensitive enzymatic assays were conducted to directly compare and quantitate their activities in oral lavage. Consistent with the proteomic results, both triosephosphate isomerase and malate dehydrogenase activities were significantly higher in high-bleeder participants compared to the low-bleeder group (Figure 3C,D).

#### Enzymatic Activities: Effects of Four Weeks of SnF_2_ Dentifrice Use

Treatment with SnF_2_ dentifrice reduced the enzymatic activities of both triosephosphate isomerase and malate dehydrogenase in oral lavage in the unhealthy gingiva participants compared to baseline (Figure 3C,D).

### 2.7. TLR-ATP Biosensor—Bacterial Structure Component Activation

Metabolic results revealed that adenosine was significantly lower in the oral lavage of high-bleeder participants compared to low bleeders at baseline (Figure 1A,B). Adenosine is a component of ATP, which stores energy in cells. Treatment with SnF_2_ dentifrice reduced levels of key citric acid cycle metabolites, succinate and α-ketoglutarate (Figure 1D) in both groups. Importantly, the top 10 proteins with the lowest *p*-values in the proteomic study included four enzymes involved in glycolysis and the citric acid cycle (Figure 3A,B). Enzymatic activities of two enzymes, triosephosphate isomerase and malate dehydrogenase, were confirmed to be higher in the oral lavage of high-bleeder participants relative to low bleeders at baseline. Further investigation into the glycolysis pathway and two related enzymes, lactate dehydrogenase and alcohol dehydrogenase, revealed that glycolysis enzymes were more abundant in the oral lavage of high-bleeder subjects than in low bleeders at baseline (Supplementary Figure S2, *p* < 0.05). These data collectively suggested that glycolysis and metabolism are elevated in the tissues of high-bleeder participants.

Collectively, the metabolomic and proteomic analyses provided converging evidence suggesting altered bioenergetics in gingivitis. Our metabolomic profiling revealed that adenosine, a key component of ATP and an important cellular energy currency, was significantly lower in the oral lavage of high-bleeder participants at baseline (Figure 1A,B). Concurrently, the proteomic analysis highlighted an upregulation of several enzymes central to glycolysis and the citric acid cycle. Specifically, four of the seven most abundant proteins in unhealthy gingiva (*p* < 0.0004) were identified as key enzymes in these pathways: aldolase A, triosephosphate isomerase, lactate dehydrogenase (LDH-H1), and malate dehydrogenase (MDHC) (Figure 3B). Further enzymatic assays directly confirmed elevated activities of triosephosphate isomerase and malate dehydrogenase in gingivitis samples (Figure 3C,D). These findings, suggesting increased activity in ATP-generating metabolic pathways alongside altered ATP-related metabolites, prompted further investigation into the direct impact of bacterial virulence factors on cellular ATP production. This led to the development of a TLR-ATP biosensor model.

To mechanistically evaluate the role of bacterial virulence factors, such as those implicated in gingivitis, in stimulating the host cellular metabolic response and ATP production, a TLR-ATP biosensor model was developed as previously described [56] This biosensor, which integrates TLR signaling components with an ATP fluorescent reporter, allowed us to directly assess the cellular bioenergetic response to pathogen-associated molecular patterns (PAMPs) identified in gingivitis. As gingival keratinocytes represent a primary innate immune barrier in the oral cavity and are involved in initial host–bacterial interactions, this cellular model facilitated a direct mechanistic understanding of how LPS and other bacterial components influence cellular metabolism and ATP dynamics. As detailed in Section 4, the biosensor was constructed using HEK-Blue™-hTLR2 cells genetically modified to express both TLR2 receptors and an ATP biosensing module.

**Figure 4:**
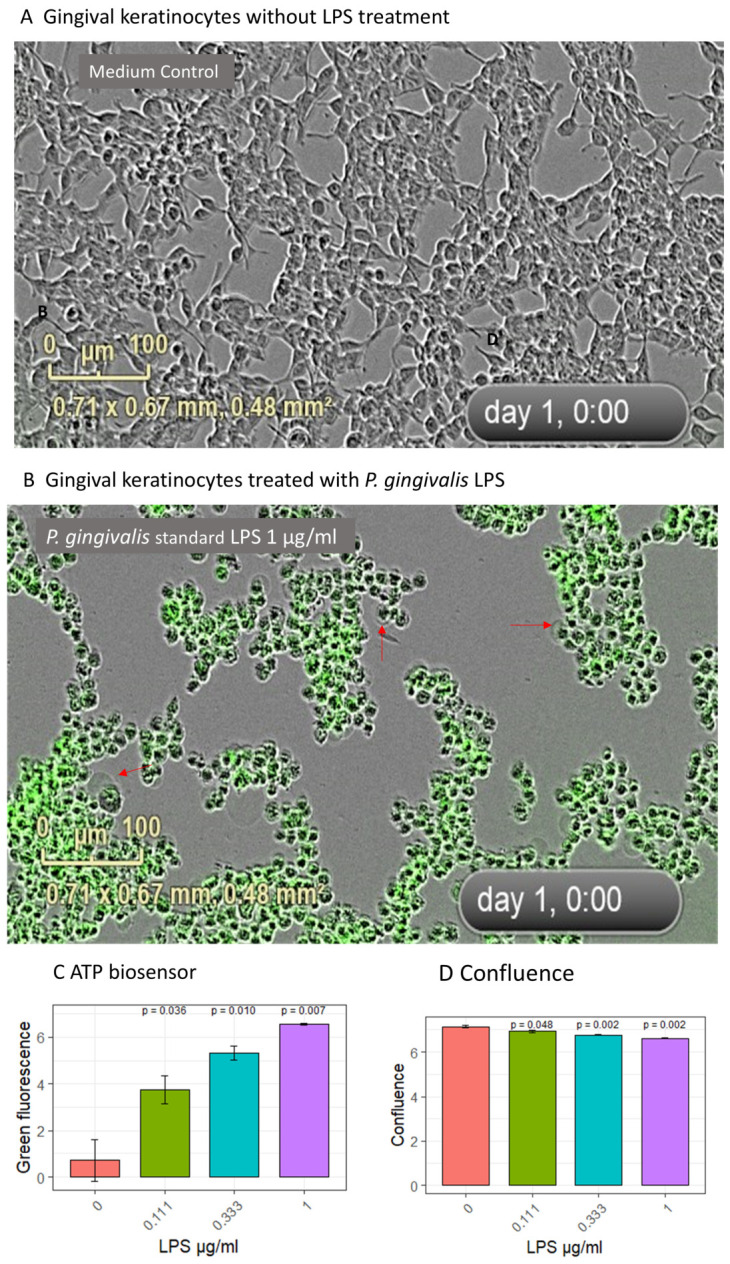
Activation of ATP biosensor by *P. gingivalis* LPS. The ATP biosensor cells were treated with (**A**). medium alone and (**B**). *P. gingivalis* LPS at 1 μg/mL in assay medium. The red arrow in the middle and to the right indicate significant swelling and formation of plasma membrane bubbles, where cells essentially exploded and released their contents into the surrounding environment (Figure 4B, indicated by the red arrow on left side). Images were taken 24 h after treatment. (**C**). Green fluorescence areas and (**D**). cellular confluence areas of cells treated with *P. gingivalis* LPS at a range of 0 to 1 μg/mL in assay medium. Results were measured from three experiments, each with at least two duplicate wells and four images from each well.

**Figure 5:**
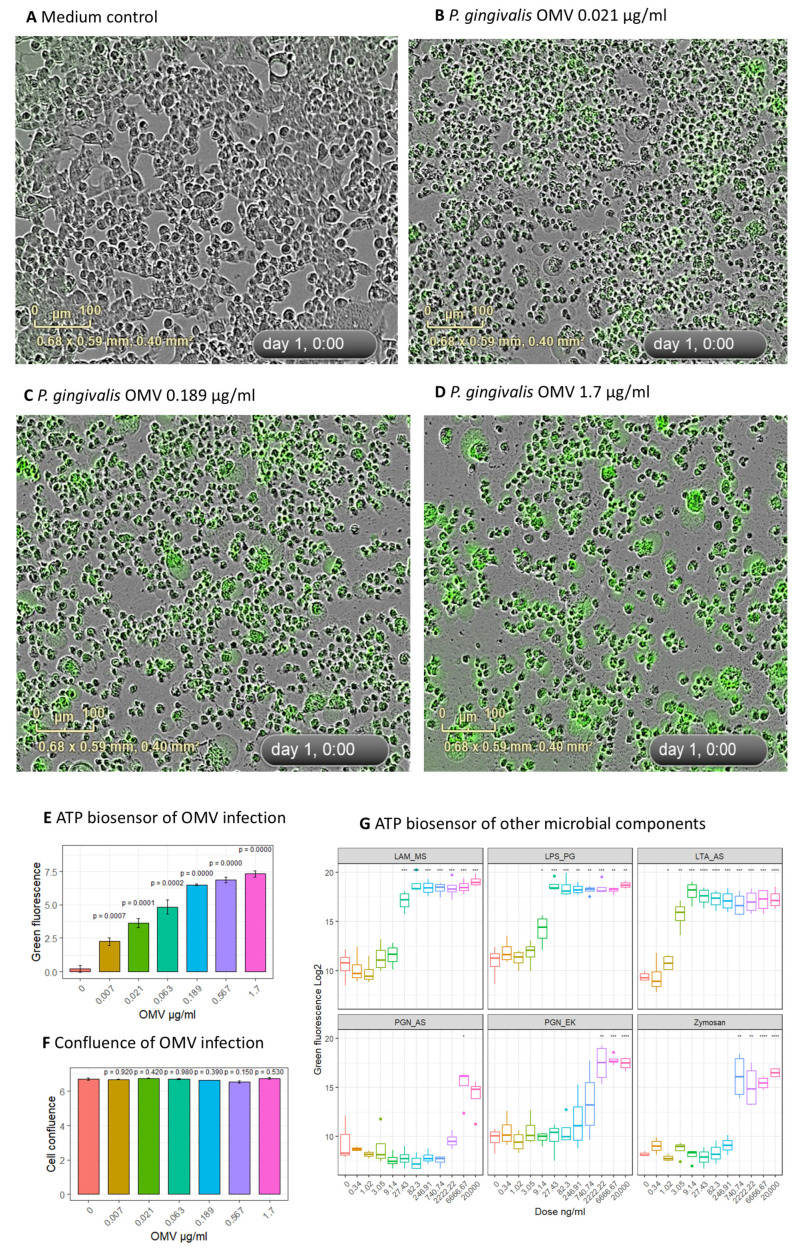
Activation of ATP biosensor by and *P. gingivalis* OMV and microbial virulence components. *P. gingivalis* OMV activated ATP-GFP biosensor in a dose-dependent manner. *P. gingivalis* OMV were applied to the TLR-ATP biosensor cells in assay medium and images were taken 24 h thereafter. (**A**). 0, (**B**). 0.021, (**C**). 0.189, and (**D**). 1.7 μg/mL *P. gingivalis* OMV. (**E**). The fluorescence areas were plotted against OMV doses, ranging from 0.007 to 1.7 μg/mL, along with a control having 0 OMV μg/mL (red bar). (**F**). Cell confluence areas were plotted against OMV doses, ranging from 0.007 to 1.7 μg/mL, along with a control having 0 OMV μg/mL (red bar). Five experiments were performed, each with three replicate wells and four images per well. Fluorescence area results were first normalized with 0.189 μg/mL OMV, and then transformed in log2. Plot and statistical calculation were performed using the ggpubr package in Rstudio (version 2026.05.0-218). (**G**) Microbial virulence activated the ATP biosensor in a dose-dependent manner. The ATP biosensor cells were plated at 5000 cells per well in a 96-well plate, and reached confluence 72 h after cell seeding. The cells were treated with microbial cell wall components in the regular growth medium, and images (green fluorescence and phase contrast) were taken at 75 h after treatments with microbial cell wall components. Each data point in boxplots of green fluorescence areas was derived from 4 experiments with 4 replicates. **** indicates *p*-value < 0.0001, *** *p*-value < 0.001, ** *p*-value < 0.01, * *p*-value < 0.05.

Bacterial virulence components (e.g., LPS, LTA) have been reported to stimulate TLR2 and TLR4 signaling pathways and as expected these substances also activated TLR-ATP biosensor cells in a dose-dependent manner (Figure 4 and Figure 5). This supports that virulence factors, including LPS and OMVs, mediate the upregulation of glycolysis and citric acid cycle activity leading to increased ATP production in keratinocytes via Toll-receptor binding.

*P. gingivalis* LPS not only induced the TLR-ATP biosensor but also promoted cell death. The affected cells exhibited significant swelling and the formation of plasma membrane bubbles (Figure 4B, indicated by the red arrow in the middle and to the right), resulting in a “bursting” appearance where the cells essentially exploded and released their contents into the surrounding environment (Figure 4B, indicated by the red arrow to the left). Additionally, LPS reduced cellular confluence (Figure 4D), confirming it caused cell death. LTA-SA, a TLR2 ligand, activated the TLR-ATP biosensor at a low concentration (1.02 ng/mL, *p* < 0.05, Figure 5). Interestingly, another TLR2 ligand from the same bacterium, peptidoglycan (PGN-SA), did not activate the TLR-ATP biosensor until it reached a much higher concentration of 6666.67 ng/mL (*p* < 0.05). Other microbial components also activated the TLR-ATP biosensor at various concentrations. These include TLR2 ligands such as LAM-MS and Zymosan, as well as TLR2 and TLR4 ligands like PGN-EK illustrated in Figure 5.

One noteworthy characteristic of the periodontal pathogen *P. gingivalis* is that this microbe releases OMVs which contain a variety of virulence factors, such as LPS and gingipains. In the experiments here, *P. gingivalis* OMVs activated the ATP biosensor in a dose-dependent manner (Figure 5).

Low background green fluorescence was observed in cells that were not treated with OMV (Figure 5A). OMV increased fluorescence intensity and areas in a dose-dependent fashion (Figure 5B–E). Cell confluence was not statistically impacted by OMV in the doses applied here for 24 h. Long exposure for about 100 h did reduce cellular confluence.

**Figure 6:**
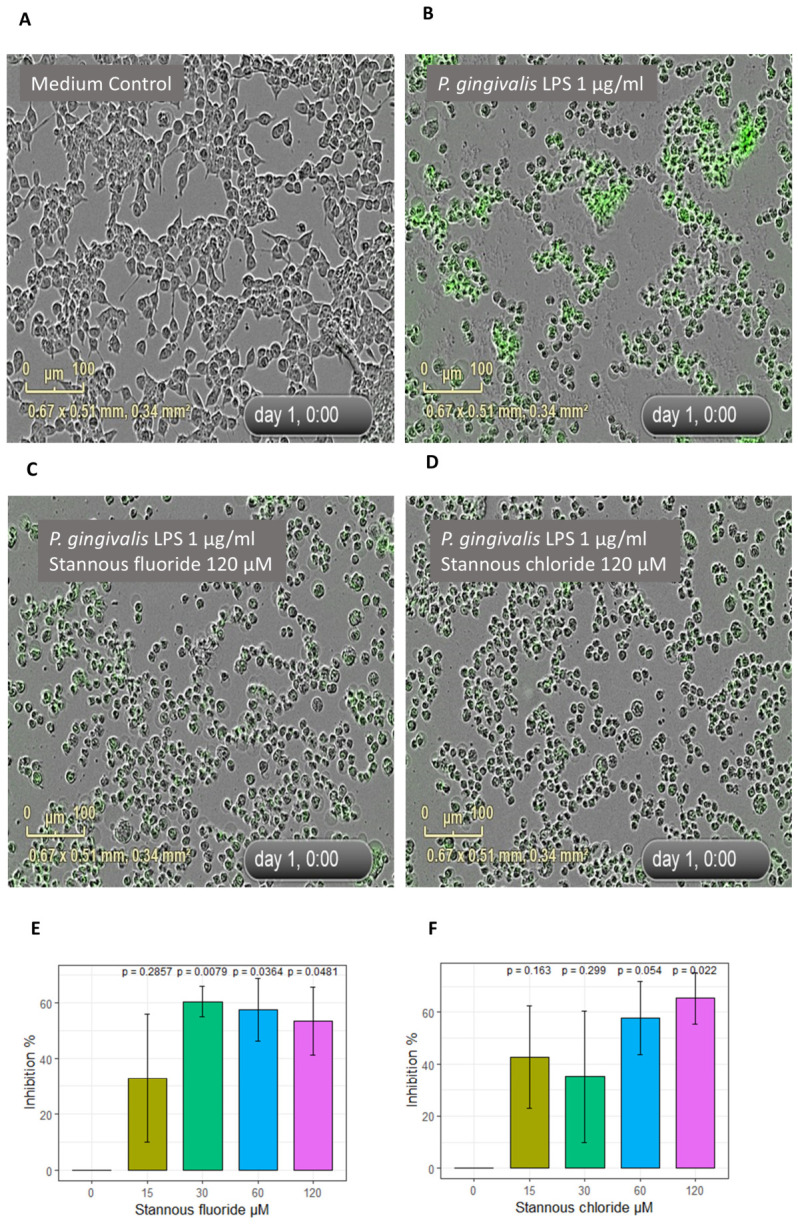
Inhibition of LPS-activated TLR-ATP GFP biosensor fluorescence by SnF_2_ and SnCl_2_. ATP biosensor cells were treated with (**A**). medium alone, (**B**). *P. gingivalis* LPS at 1 μg/mL, (**C**). *P. gingivalis* LPS at 1 μg/mL and 120 μM SnF_2_, and (**D**). *P. gingivalis* LPS at 1 μg/mL and 120 μM SnCl_2_ in assay medium. Images captured 24 h post-treatment. Fluorescence areas were plotted against (**E**). SnF_2_ and (**F**). SnCl_2_ doses. Three independent experiments were conducted, each with at least three replicate wells and four images per well. Bar plots and statistical analyses were performed using the ggpubr package in RStudio.

Previously, it was demonstrated that SnF_2_ bound LPS in a one-to-one molar ratio and prevented LPS from binding to TLRs in HEK293 cells. Additionally, SnF_2_ disrupted LPS-induced TLR signaling in both TLR2-based and TLR4-based reporter gene assays. Lastly, SnF_2_ was shown to inhibit the production of proinflammatory cytokines in human primary monocytes. In the current experiments, SnF_2_ was added to the TLR-ATP biosensor cells simultaneously with the LPS. The results showed that SnF_2_ prevented LPS from activating the ATP biosensor (Figure 6). The green fluorescence intensity was brighter, and the fluorescence areas were larger in the LPS alone (Figure 6B) compared to those treated with either SnF_2_ or stannous chloride (Figure 6C,D).

**Figure 7:**
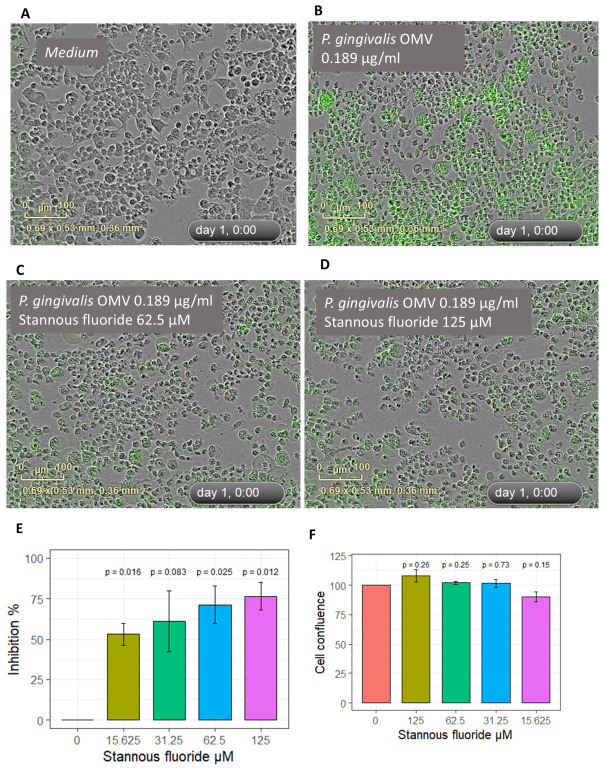
SnF_2_ inhibited OMV-activated TLR-ATPGFP biosensor fluorescence. (**A**). Green fluorescence was not detectable if no OMV was applied at 24 h. (**B**). OMV 0.189 μg/mL along, (**C**). OMV 0.189 μg/mL SnF_2_ and 62.5 μM SnF_2_, (**D**). OMV 0.189 μg/mL SnF_2_ and 125 μM SnF_2_. (**E**). The green fluorescence areas were measured and compared with control which did not have any SnF_2_. *p*-value in each bar represents comparison between itself and 0 μM SnF_2_. (**F**). Cell confluence represents areas which cells covered. Three experiments were carried out, each with two replicate wells, each well with four image sites. Observations at 24 h after treatments are shown in the images and bar plots. The red bar represents 0 μM SnF_2._ The green fluorescence area and confluence area were taken in the same sites.

Finally, SnF_2_ was examined for its effects on OMV activation of TLR-ATP biosensor cells. Almost no green fluorescence was emitted in the TLR-ATP biosensor cells if no OMV was applied at 24 h (Figure 7A). In contrast, many cells exhibited fluorescence with the addition of the OMV (Figure 7B). The green fluorescence was reduced in areas and intensity if Sn was included (Figure 7C,D). SnF_2_ inhibited OMV-induced activation of the TLR-ATP biosensor at concentrations of 15, 60 and 120 mM at 24 h (Figure 7E). However, neither SnF_2_ nor OMV had any deleterious effect on the observed cellular confluence at 24 h (Figure 7F).

## 3. Discussion

Oral lavage samples underwent multi-omic analysis, including comprehensive metabolomics (Metabolon Inc., Metabolon, Morrisville, NC, USA), a 40-plex proteomic cytokine assay, aptamer-based proteomics (SomaLogic Inc., Boulder, CO, USA) on a subset, and two direct enzyme evaluations. However, sample sizes were relatively small: 20 for baseline proteomics (split equally between low and high bleeding) and 76 across baseline and week 4 metabolomics. Consequently, these interpretations and hypotheses should be considered preliminary. In metabolomic analysis, a number of molecules were identified showing significant pronounced differences. These included adenosine, guanosine, 4-Hydroxyphenylpyruvate, 5′-Methylthioadenosine (MTA), oxidized cys-gly, 3-Hydroxyisobutyrate (3-HIB) and indolepropionate. Both adenosine and guanosine occur widely in nature in the form of diverse derivatives, such as ATP and guanosine triphosphate. Adenosine plays a crucial role in bioenergetics, particularly in energy transfer within cells. 4-Hydroxyphenylpyruvate is an intermediate in the metabolism of the amino acids tyrosine and phenylalanine. It is converted to homogentisic acid, which is one of the precursors to ochronotic pigment. It is produced in human cells as well as in bacteria. 5′-Methylthioadenosine (MTA) is an S-methyl derivative of adenosine and a toxic byproduct of cellular processes involving S-adenosylmethionine. Oxidized cys-gly is a breakdown byproduct of oxidized glutathione, indicative of oxidative stress. 3-Hydroxyisobutyrate (3-HIB) is a catabolic intermediate of the branched-chain amino acid valine. Lastly, indolepropionate is a breakdown byproduct of tryptophan, mediated primarily by bacteria, indicating high bacterial enzymatic activity in high bleeders at baseline. The combined metabolite concentration differences in oral lavage between healthy and unhealthy gingiva suggested that the unhealthy gingiva group experienced metabolic disturbances in bioenergetics (decrease in adenosine), accumulated more toxic byproduct (higher 5′-methylthioadenosine), and exhibited evidence of elevated oxidative stress (increased oxidized cys-gly) and an expanded bacterial load (higher indolepropionate). The final observation in metabolomic analysis of oral lavage was elevated levels of CRP. CRP functions as a sensor and activator for the innate immune response by binding, opsonizing, and inducing the phagocytosis of bacteria and apoptotic cells.

Turning to proteomics analysis, proinflammatory cytokines (IFNγ, IL-6, VEGF, IL-1β,TNFα) are often reported to increase in the saliva of patients with gingivitis and periodontitis [57]. These results were confirmed in the 40-plex analysis carried out in this study comparing baseline levels between healthy and unhealthy gingiva in oral lavage. IL-1β (*p* = 0.08254), TNFα (*p* = 0.08315), ICAM-1 (*p* = 0.0723) and IL-1α (*p* = 0.0518) were directionally higher in the unhealthy gingiva group in the 40-plex assay (Figure 3). CRP, an inflammatory biomarker produced primarily in the liver, was likewise confirmed to be elevated in the oral lavage of the unhealthy gingiva group (*p* = 0.0165; Figure 3) as was observed in the metabolomic analysis. However, it remains to be investigated whether the CRP was produced locally or systemically. Aptamer-based proteomics carried out with Somologic Proteomics revealed a striking abundance of enzymes associated with glycolysis and citric acid cycles in oral lavage of unhealthy gingiva participants (Figure 4). Four proteins, including aldolase, malate dehydrogenase, lactate dehydrogenase and triosephosphate isomerase were among the top 7 proteins which exhibited the most significant differences among the 1310 proteins analyzed. In fact, a number of other enzymes associated with glycolysis were also increased in oral lavage of participants with unhealthy gingiva (see Supplementary Figure S2). They included phosphoglycerate kinase I, pyruvate kinase PKM, glyceraldehyde-3-phosphate dehydrogenase (GAPDH), alcohol dehydrogenase, phosphoglycerate mutase I, and pyruvate phosphate kinase I. Malate dehydrogenase catalyzes conversion of malate into oxaloacetate. The three substrates preceding malate in the biochemical process are fumarate, succinate and α-ketoglutarate in the citric acid cycle. Both succinate and α-ketoglutarate were decreased in oral lavage after treatment with SnF_2_ dentifrice for 4 weeks in the unhealthy gingiva group (Figure 2).

Considering the metabolomic results together with the proteomics results, both glycolysis and citric acid cycle reactions were elevated in the unhealthy gingiva group. The combined analyses supported that four of the top seven proteins based on *p*-values < 0.0004 were enzymes in the glycolysis pathways (aldolase A, triosephosphate isomerase, lactate dehydrogenase) and citric acid cycle (malate dehydrogenase), which increased in the oral lavage of the unhealthy gingiva group. Confirmation of the metabolomic and proteomic findings were then obtained by specific analysis of malate dehydrogenase and triosephosphate isomerase activities in oral lavage aliquots. Both enzyme activities were elevated in participants with unhealthy gingiva, and, conspicuously, the treatment with SnF_2_ dentifrice reduced their respective activities. These observations support that ATP production is elevated during inflammation, aligning with previous research, indicating that succinate acts as an inflammatory signal that induces IL-1β through HIF-1α [58]. LPS induces a shift in cellular metabolism from oxidative phosphorylation to glycolysis, which leads to increased expression of genes in the glycolysis pathway. These metabolic alterations directly correlate with the expression profiles of specific metabolites. Notably, LPS significantly increases succinate levels, a crucial intermediate in the tricarboxylic acid pathway. Additionally, LPS enhances the succinylation of various proteins, further promoting IL-1b production during inflammation. The metabolic and proteomic findings from the clinical study, combined with the published literature, informed the development of the ATP biosensor cell line to test the hypothesis that bacterial virulence factors (e.g., LPS, etc.) mediate the upregulation of cellular bioenergetic pathways leading to ATP production as part of the initial host response top host–bacterial interactions during infection.

The ATP biosensor model was developed by genetically transfecting the genome of HEK-Blue™-hTLR2 cells with TLR2 signaling components in collaboration with TempoBiosciences Inc. This cellular model facilitated a mechanistic understanding of the effects of LPS and other bacterial components on cellular metabolism. *P. gingivalis* LPS not only energized the TLR-ATP biosensor but also induced cell death. Additionally, LPS reduced cellular confluence, confirming it caused cell death. LTA-SA activated the TLR-ATP biosensor at low concentrations (1.02 ng/mL) while another TLR2 ligand from the same bacterium, peptidoglycan (PGN-SA), did not activate the ATP biosensor until applied at 6600X the concentration. Other microbial components also activated the ATP biosensor at various concentrations. One of the noteworthy characteristics of the periodontal pathogen *P. gingivalis* is that this microbe releases OMV, which contains a variety of virulence factors, such as LPS and gingipains. In the experiments here, *P. gingivalis* OMV activated the TLR-ATP biosensor in a dose-dependent manner. Experiments were then undertaken to assess the potential of SnF_2_ to suppress ATP activation in biosensor cells. In the current experiments, SnF_2_ was added to the TLR-ATP biosensor cells simultaneously with the LPS. The results showed that SnF_2_ prevented LPS from activating the TLR-ATP biosensor. SnF_2_ was additionally examined for its effects on OMV potentiation of TLR-ATP biosensor cells. SnF_2_ inhibited OMV-induced activation of the TLR-ATP biosensor at concentrations of 15, 60 and 120 mM at 24 h. Neither SnF_2_ nor OMV had any deleterious effect on the observed cellular confluence at 24 h.

**Figure 8:**
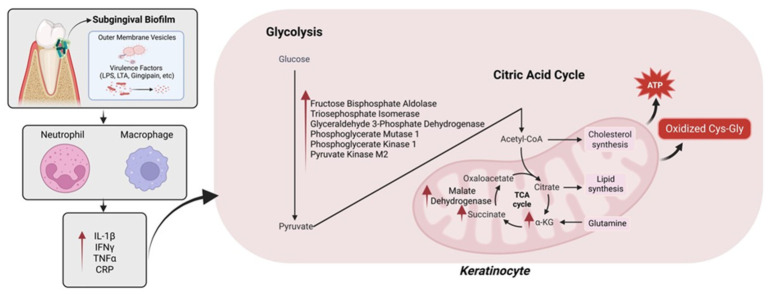
Hypothesized model of activation of ATP production during provocation of inflammation. Subgingival plaque generates virulence factors which appear to induce cytokine production (notably IL-1β), potentially leading to upregulation of the glycolytic pathway and portions of the citric acid cycle. This increase in central metabolic pathways may result in elevated ATP and free radical concentrations. Extracellular ATP is lethal to animals in experimental models, inflames cytokine production, and promotes cellular death. Created in https://BioRender.com.

A conceptual model (Figure 8) is herein presented to synthesize our metabolomic and proteomic data, offering a potential framework for understanding the augmented glycolysis and citric acid cycle processes observed in individuals with gingivitis. It is posited that with the maturation of subgingival biofilms under conditions of inadequate oral hygiene, an increased influx of OMV-associated and free virulence factors—such as LPS, peptidoglycans, and LTA—into the subgingival gum tissue appears to instigate heightened production of proinflammatory cytokines. This process is believed to unfold through an immune cell-dependent mechanism, initiated by the recognition and capture of PAMPs associated with these elevated virulence factors. The ensuing cytokine response, exemplified by IL-1β, may then contribute to the stabilization of transcription factor HIF-1α, leading to a potential upregulation in the expression and translation of key enzymes within glycolysis and the citric acid cycle. This could, in turn, result in elevated ATP production, alongside a concomitant increase in oxidative stresses, as evidenced by an uptick in oxidized Cys-Gly. However, it is important to acknowledge that the intricate biological reasons for increased ATP levels during inflammation remain an active area of research, and the diverse extracellular roles of ATP in various systemic conditions, such as heart disease, cancer, and diabetes, are subjects of continuous and extensive investigation [57,59,60,61,62,63,64,65]. A potential functional role for this heightened ATP production could be to support the cellular reparative or defensive responses to injury induced by the virulence factors (e.g., OMVs) associated with the detected PAMPs.

## 4. Materials and Methods

### 4.1. Clinical Study Background

This study carried out a supplemental analysis of oral lavage samples which were collected as part of a published clinical study examining the efficacy and mechanisms of gingivitis reductions provided by 0.454% SnF_2_ dentifrice [39]. Briefly, forty participants meeting the inclusion/exclusion criteria were recruited for study enrollment. Clinical examinations identified 20 participants as a healthy gingiva cohort, with 3 or fewer bleeding sites and all periodontal pockets measuring less than or equal to 2 mm deep. Another 20 participants were qualified as an unhealthy gingiva cohort, with clinical examinations showing greater than 20 bleeding sites and at least 3 pockets measuring greater than or equal to 3 mm, but not deeper than 4 mm. Following collection of initial samples (see below) all participants (both high and low bleeders) were provided with 0.454% SnF_2_ dentifrice (Crest Pro-Health Clinical Gum Protection Toothpaste, Procter & Gamble, Cincinnati, OH, USA) and a soft manual toothbrush (Oral-B Indicator, Procter & Gamble, OH, USA) for use in the 4-week treatment phase of the study. Samples collected for analysis included supra- and subgingival plaque (baseline, 2 and 4 weeks of treatment) and morning oral lavage (5 collections on 5 consecutive days preceding baseline, week 2 and week 4). Oral lavage samples were stored in the freezer in participants’ homes prior to surrendering them at their scheduled clinical visits.

### 4.2. Oral Lavage Samples

Oral lavage samples were collected upon waking (one per participant) by rinsing with 4 mL of water for 30 s and then expectorating the contents into a centrifuge tube. Oral lavage samples comprise secretions from salivary glands, fluid exudate expressed from gingival crevicular areas, and metabolites derived from oral microbes, epithelial cells, and infiltrated immune cells. The oral lavage sample collected upon first waking in the morning is preferentially devoid of dilution from eating and drinking and is a prerequisite for metabolomic studies [66]. These samples were frozen at home until they were brought to the clinical site (Salus Research, Inc., Ft. Wayne, IN, USA) in a cold pack to keep the sample in a frozen state. A pair of samples, consisting of baseline and week 4 samples, was collected from each subject, including 20 from the healthy gingiva group and 18 from the unhealthy gingiva group. Upon submission, oral lavage samples were kept frozen at −20 °C and transported under dry ice to the P&G Mason Business & Innovation Center and stored at −80 °C until analysis. For the analysis reported in this paper, retained frozen samples from the original clinical study were defrosted, vortexed, and centrifuged at 6654× *g* for 30 min to separate the supernatant from the solidified pellet. The supernatant was collected and aliquoted into 125 μL or 1 mL portions for each analysis. All samples underwent two freeze–thaw cycles prior to analysis. The first freeze occurred a home, while the second took place in the laboratory after processing and aliquoting on ice for each analysis. The analyses included metabolomics, biomarker immunoassays, proteomics on SomaScan^®^ platform, and enzymatic assays outlined below.

### 4.3. Metabolomic Analysis

All available samples at baseline and week 4 were extracted and analyzed using Metabolon’s global biochemical profiling platforms (Metabolon, Morrisville, NC, USA) following standard procedures in 2015 [55]. Briefly, samples (125 μL each) of 18 healthy and 20 unhealthy subjects at baseline and week 4 were extracted and aliquoted for analysis on the LC-MS/MS platforms. Proprietary software was used to match ions to an in-house library of standards for metabolite identification and quantitation by peak area integration. Data normalization and statistical analyses were performed by statistical professionals at Metabolon using analysis of variance (ANOVA) and a two-sided *t*-test. The differences were compared between high and low bleeders at baseline and week 4 using t-tests. Changes within the high-bleeder group or within the low-bleeder group were compared using paired *t*-tests. q-values were calculated to control the false discovery rate (FDR). A q-value indicates the minimum FDR at which a particular test may be considered significant. For example, if a q-value is 0.05, this means that 5% of the tests that are declared significant at this threshold are expected to be false discoveries. The statistical analyses were performed on natural log-transformed data.

### 4.4. Cytokine Analysis via Biomarker 40-Plex

The V-PLEX Human Biomarker 40-Plex Kit from Meso Scale Diagnostics (Rockville, MD, USA) was used to measure protein biomarkers in oral lavage samples. The 40-plex kit includes proteins representing biomarkers involved in cardiovascular, immunological, and inflammatory functions, as well as cytokines and chemokines. These biomarkers include CRP, Eotaxin, Eotaxin-3, FGF (basic), GM-CSF, ICAM-1, IFN-γ, IL-1α, IL-1β, IL-2, IL-4, IL-5, IL-6, IL-7, IL-8, IL-10, IL-12/IL-23p40, IL-12p70, IL-13, IL-15, IL-16, IL-17A, IP-10, MCP-1, MCP-4, MDC, MIP-1α, MIP-1β, PlGF, SAA, TARC, Tie-2, TNF-α, TNF-β, VCAM-1, VEGF-A, VEGF-C, VEGF-D, and VEGFR-1/Flt-1. The assay was performed according to the manufacturer’s instructions. Briefly, the binding antibody was deposited in specific spots on the assay plate. Oral lavage samples (50 μL) were added to the assay plate, which was then covered with an adhesive seal and incubated at room temperature with shaking for 2 h. The plate was washed three times, and the detection antibody was added. After another wash, the plate was filled with 150 μL of 2× Read Buffer T. Finally, the plate was read using the Meso Sector S 600MM (Meso Scale Diagnostics). The difference between high and low bleeders at baseline was compared by using Welch’s Two-Sample *t*-test. An adjusted *p*-value was calculated using the Benjamini–Hochberg procedure.

### 4.5. Proteomic Analysis

The oral lavage samples were initially categorized into high and low bleeders based on clinical observations, including gingival bleeding and inflammation at baseline [39]. Metabolic profiling was conducted using Metabolon procedures, which facilitated the construction of a dendrogram that grouped the oral lavage samples into two distinct categories (as illustrated and described in Figure 1). The upper group primarily consisted of samples from high bleeders, while the lower group included samples from low bleeders.

For proteomic analysis, samples were selected based on two criteria: clinical classification and cluster grouping. Specifically, 10 samples were chosen from the high-bleeder category, which were clustered in the upper section of Figure 1, with the exception of one sample that was incorrectly classified in the upper region from the low group due to a reading error. These samples are referred to as “high bleeders” in the proteomic results. Conversely, another 10 samples were selected from the low bleeder category, residing in the lower cluster, and are labeled as “low bleeders” in the proteomic results.

SomaLogic (an aptamer-based array) and mass spectrometry (MS) represent two fundamentally different approaches to proteomics. SomaLogic acts as a high-throughput, targeted “search engine” for known proteins, whereas mass spectrometry provides unbiased, deep-coverage discovery identifying both known proteins and unknown structural variants. Proteomic analysis was conducted in 2016 at SomaLogic Inc. (Boulder, CO, USA) using their aptamer-based SomaScan^®^ Platform, which quantifies 1310 protein targets [67,68,69]. While SomaScan functions as a high-throughput, targeted ‘search engine’ for known proteins, it fundamentally differs from mass spectrometry (MS)-based proteomics (typically LC-MS/MS). MS-based approaches offer unbiased, deep-coverage discovery, capable of identifying both known proteins and novel structural variants, and can also detect post-translational modifications like phosphorylation and glycosylation. SomaScan, conversely, is sensitive and reproducible but is limited to detecting only those proteins it is specifically programmed to target. The aptamer reagent used in this analysis is a single-stranded DNA-based molecule that specifically binds to target proteins. The Aptamer-based Proteomic Platform measures proteins directly from oral lavage samples using a multi-step capture, release, and re-capture enrichment process. The difference between high and low bleeders at baseline was compared by using Welch’s Two-Sample *t*-test. An adjusted *p*-value was calculated using the Benjamini–Hochberg procedure.

### 4.6. Enzymatic Assays

Both the triosephosphate isomerase activity colorimetric assay kit and the malate dehydrogenase activity kit were purchased from Sigma (St. Louis, MO, USA). Assays were performed in 2017 according to the manufacturer’s instructions. Briefly, oral lavage samples (50 μL) were first added to wells of a 96-well plate, followed by the addition of the reaction mix (50 μL), which included assay buffer, substrate, and enzymes. The samples and reaction mix were then incubated at 37 °C. Absorbance at 450 nm was measured. NADH standards were included in the assay. The amount of NADH (nmole/mL) in each sample at minute 30 was calculated based on the standard curve generated using OD 450 measurements of NADH standards in each assay.

### 4.7. Cell Line Construction and Assay Development for TLR-ATP Biosensor

In the aforementioned analyses, four of the top seven proteins (based on *p*-values < 0.0004) that were elevated in the oral lavage of the high-bleeding group were enzymes in the glycolysis pathways (aldolase A, triosephosphate isomerase, lactate dehydrogenase) and citric acid cycle (malate dehydrogenase). These results suggested over-production of ATP in participants in the high bleeding group. Enzymatic assays confirmed the proteomic finding that malate dehydrogenase and triosephosphate isomerase activities were higher in the high bleeding oral lavage samples. These collective results prompted the development of a TLR-ATP biosensor cell model for further study. The cell line was constructed in collaboration with TempoBiosciences (San Francisco, CA, USA) as previously described [56]. This cell line contains two specific elements in addition to the usual cellular machinery and organelles: the TLR2 receptor and the ATP biosensor. HEK-Blue™-hTLR2 cells, purchased from Invivogen (San Diego, CA, USA), were established by co-transfection of the human TLR2 receptor and other genes into HEK293 cells. The ATP biosensor, TempoATP™, a fluorescent biosensor, was incorporated into the HEK293 TLR2 receptor cell line. The biosensor assay offers fast kinetics (resolution in seconds) for measuring real-time cellular ATP metabolism and oxidative phosphorylation, which are directly associated with mitochondrial health and cellular bioenergetics. The amino acid sequence of the TempoATP™ Biosensor is a fusion protein consisting of an ATP-binding peptide sequence and a green fluorescent protein sequence (GFP) (Supplementary Table S4) (from Fiorello et al. [69]). This fusion TLR-ATP biosensor protein is then constitutively expressed in the cells. As intracellular ATP levels increase, ATP binds to the biofusion sensor, triggering a conformational change that alters the structure of the GFP domain. Consequently, the TLR-ATP biosensor protein changes its shape, enhancing the fluorescence of the GFP. The fluorescence intensity reflects the presence of ATP, becoming brighter or dimmer depending on the binding event of the fusion protein to ATP. The change in fluorescence was measured in real time using an IncuCyte^®^ Live-Cell Analysis System (IncuCyte Zoom, Sartorius AG, Ann Arbor, MI, USA) with an excitation wavelength of 519 nm and an emission wavelength of 535 nm (green fluorescence).

### 4.8. Experiments with TLR-ATP Biosensor Including Effects of Various Virulence Factors and Inhibition by SnF_2_

Microbial virulence factors evaluated include individual molecules (e.g., LPS) as well as OMVs of *P. gingivalis*. The individual virulence molecules were purchased directly from InvivoGen (San Diego, CA, USA), including LPS from *P. gingivalis* (LPS-PG), lipoarabinomannan from *Mycobacterium smegmatis* (LAM-MS), LTA from *Bacillus subtilis* (LTA-BS), peptidoglycans from *Escherichia coli* K12 (PGN-EK), peptidoglycans from *Staphylococcus aureus* (PGN-SA), and zymosan, an insoluble cell wall preparation from *Saccharomyces cerevisiae* (Zymosan). OMVs were prepared and isolated as previously described [70]. Briefly, *P. gingivalis* (ATCC catalog #33277, American Type Culture Collection, Manassas, VA, USA) was cultured in MTGE media (Anaerobic Enrichment Broth, Anaerobe Systems, Morgan Hill, CA, USA), and the medium was collected after 72 h by centrifugation. OMVs were secreted by *P. gingivalis* into the MTGE media. The medium was then filtered through 0.45 μm pore PVDF membranes to remove cell debris. The volume of the medium was reduced by filtration using a tangential flow filtration Minimate TFF System (PALL Life Sciences, Port Washington, NY, USA) with filter capsules of molecular weight cutoff 100 kD at 40 psi. The retentate from the filtration was centrifuged at 140,000× *g* for 1 h at 4 °C using an SW32 swinging bucket rotor on a Beckman XL-100K Ultracentrifuge (Beckman Coulter, Atlanta, GA, USA) to separate the OMV pellet from the supernatant. The pellets were resuspended in dPBS buffer (1× Dulbecco’s Phosphate Buffered Saline (dPBS): Life Technologies, Grand Island, NY, USA) and centrifuged at 200,000× *g* for 1 h at 4 °C (using an SW41 swinging bucket rotor) to yield a standard OMV preparation. The OMVs were resuspended in endotoxin-free water, aliquoted into 0.5 mL Eppendorf tubes, and stored at −80 °C. OMVs were quantified using the Bradford assay to estimate protein amounts. The amount of OMV used in experiments was based on the protein concentrations in the OMV preparation (μg/mL).

TLR-ATP biosensor cells (500,000) were seeded and maintained in 15 mL of growth medium, consisting of DMEM supplemented with 10% fetal calf serum, 1× HEK-Blue™ Selection (a solution combining several selective antibiotics), 50 μg/mL Normocin, and 1 μg/mL Puromycin (Invitrogen, San Diego, CA) in T75 flasks for three days at 37 °C, 5% CO_2_, and 95% humidity. For treatment, wells of a 96-well plate were seeded with 7500 cells/well in 100 µL of growth medium. The cells were incubated for 72 h at 37 °C, 5% CO_2_, and 95% humidity until day 4. On day 4, the medium (100 µL) was changed to the assay medium, which was HEK-Blue™ Detection medium (Invitrogen, San Diego, CA, USA) containing microbial virulence factors, including LPS-PG, LAM-MS, LTA-BS, PGN-EK, PGN-SA, Zymosan, and *P. gingivalis* OMVs. Microbial virulence factors were first resolved or mixed with the assay medium. The plate was placed into an IncuCyte^®^ instrument (an IncuCyte^®^ Live-Cell Analysis System, IncuCyte Zoom, Sartorius AG, Ann Arbor, MI, USA). Images were taken every one to three hours with an objective at 10× or 20×, channel selection for phase contrast, and green scan type with 3 to 4 images per well. The results were processed and analyzed using the IncuCyte^®^ S3 Software included with the instrument.

### 4.9. Statistical Analyses

Results were organized and analyzed using RStudio Version 2024.12.0+467. ANOVA was first performed to analyze the entire data set. When significant variations were found, a t-test was used to assess the differences between groups using the stat_compare_means function in the ggpubr package for bar plots. For line plots and some bar plots, ANOVA was first performed, and differences between treatment groups were compared using pairwise *t*-tests. Volcano plots, heatmaps, and dendrograms were constructed using the ggplot, factoextra, cluster, and gplots packages in RStudio.

## 5. Conclusions

Taken together, the present study results support that gingivitis is not just an inflammatory and oxidative disease, but also involves an upregulation of bioenergetic processes with elevated enzymatic activities in glycolysis and citric acid cycles.

## Data Availability

The data that support the findings of this study are not publicly available due to their proprietary nature but may be available from the corresponding author upon reasonable request.

## Supplementary Materials

The following supporting information can be downloaded at https://www.mdpi.com/article/10.3390/ijms27125316/s1.
