# Supplementary material for: Gingivitis Pathogenesis Involves Upregulation of Glycolysis and Citric Acid Cycle Activity Mediated by Bacterial Virulence Factors
Source: Int J Mol Sci. 2026 Jun 12;27(12):5316. doi: 10.3390/ijms27125316 (PMC13299710; doi:10.3390/ijms27125316)
Supplement: Supplementary file 1 [file ijms-27-05316-s001.zip › Supplemental Figures.pdf]

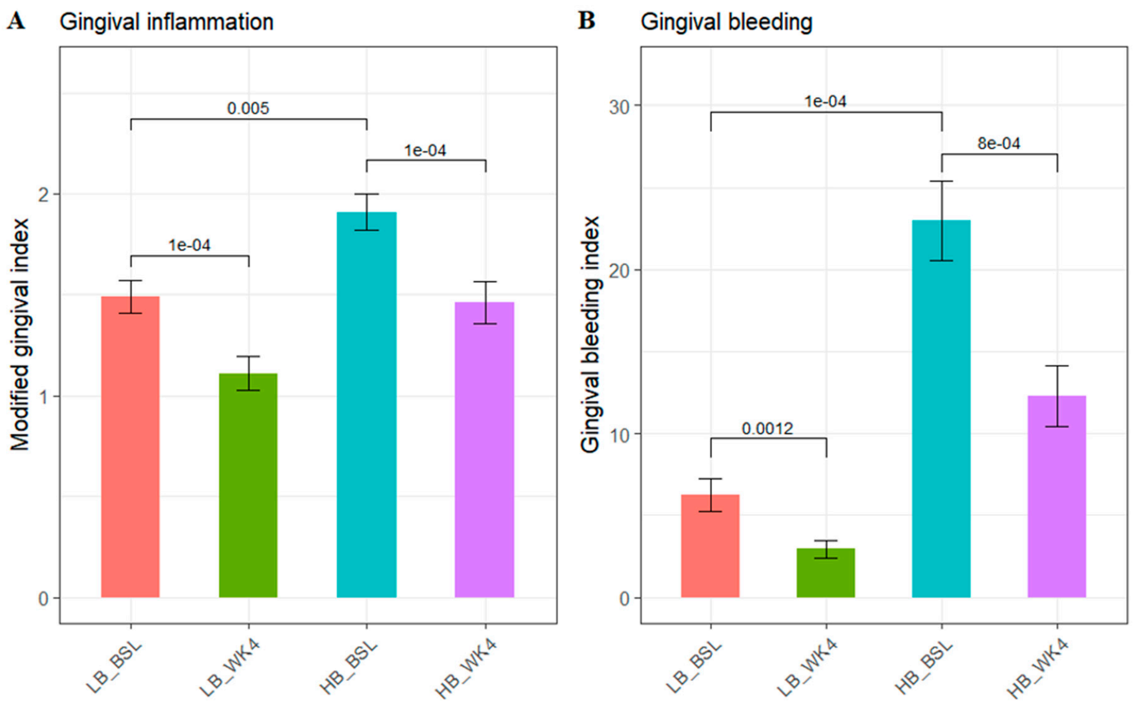

Supplementary Figure S1: Clinical symptoms of gingival inflammation and bleeding were improved with SnF_2 dentifrice treatment over four weeks. This figure shows clinical findings for gingival inflammation and bleeding assessments at baseline and after four weeks of intervention with the SnF_2 dentifrice. At baseline, groups stratified as high and low bleeding were statistically significantly different in both bleeding sites and gingival inflammation. Intervention with the SnF_2 dentifrice produced marked and statistically significant reductions in gingival bleeding and inflammation for both high bleeding and low bleeding cohorts at Week 4. (Figure was constructed based on the results of Klukowska M, Haught JC, Xie S, Circello B, Tansky CS, Khambe D, Huggins T, White DJ. Clinical Effects of Stabilized Stannous Fluoride Dentifrice in Reducing Plaque Microbial Virulence I: Microbiological and Receptor Cell Findings. *J Clin Dent*. 2017 Jun;28(2):16-26.)

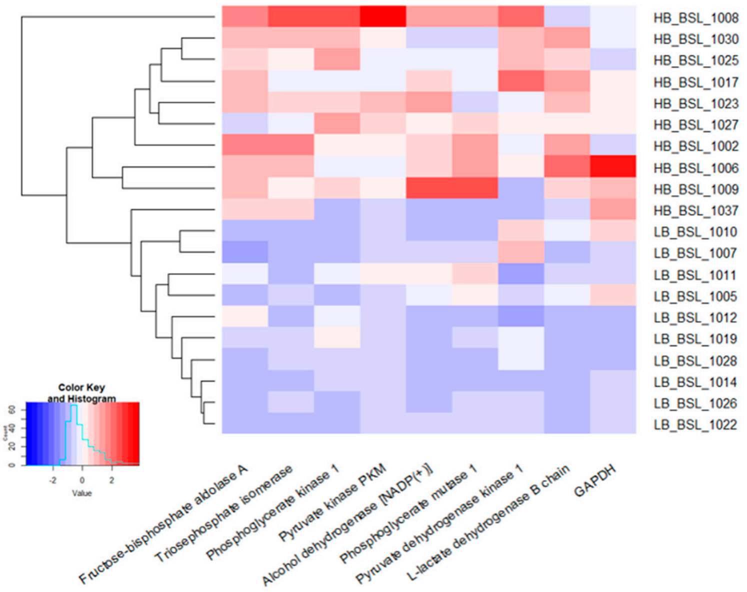

Supplementary Figure S2. Comparison of glycolysis enzymes in oral lavage between low and high bleeding participants at baseline ($P < 0.05$). Proteins were analyzed and quantified in SomoLogic Inc.
